# Supplementary material for: An alternatively spliced, non-signaling insulin receptor modulates insulin sensitivity via insulin peptide sequestration in C. elegans
Source: eLife. 2020 Feb 25;9:e49917. doi: 10.7554/eLife.49917 (PMC7041946; doi:10.7554/eLife.49917)
Supplement: Supplementary file 1. [file elife-49917-supp1.docx]

## Summary data for all replicates of lifespan experiments.

| **Genotype** | **Trial** | **Median survival** | **Deaths**  **(censored)** | **P value vs control** |
| --- | --- | --- | --- | --- |
| N2 | 1 | 14 | 70(3) |  |
| + native DAF-2B OE |  | 21 | 122(13) | P<0.0001 |
| N2 | 2* | 15 | 77(3) |  |
| + native DAF-2B OE |  | 20 | 101(13) | P<0.0001 |
| N2 | 1 | 16 | 97(5) |  |
| + neuronal DAF-2B OE |  | 24 | 119(16) | P<0.0001 |
| *daf-2(e1368)* |  | 20 | 93(7) | P<0.0001 |
| N2 | 2* | 12 | 101(1) |  |
| + neuronal DAF-2B OE |  | 18 | 176(5) | P<0.0001 |
| *daf-2(e1368)* |  | 22 | 94(11) | P<0.0001 |
| N2 | 1 | 13 | 85(3) |  |
| + hypodermal DAF-2B OE |  | 16 | 86(4) | P<0.0001 |
| N2 | 2* | 15 | 66(1) |  |
| + hypodermal DAF-2B OE |  | 18 | 84(6) | P<0.0001 |
| N2 | 1 | 15 | 73(3) |  |
| + muscle DAF-2B OE |  | 20 | 136(7) | P<0.0001 |
| N2 | 2* | 12 | 99(11) |  |
| + muscle DAF-2B OE |  | 17 | 161(17) | P<0.0001 |
| N2 | 1 | 14 | 64(1) |  |
| + intestine DAF-2B OE |  | 21 | 118(7) | P<0.0001 |
| N2 | 2* | 12.5 | 88(2) |  |
| + intestine DAF-2B OE |  | 19 | 144(11) | P<0.0001 |
| *daf-2b(Δ)* control | 1 | 13 | 165(30) |  |
| *daf-2b(Δ)* |  | 11 | 176(24) | P<0.05 |
| *daf-2c(Δ)* control |  | 12 | 176(24) |  |
| *daf-2c(Δ)* |  | 11 | 167(25) | P<0.05 |
| *daf-2b(Δ)* control | 2 | 11 | 161(29) |  |
| *daf-2b(Δ)* |  | 11 | 179(21) | ns |
| *daf-2c(Δ)* control |  | 11 | 189(17) |  |
| *daf-2c(Δ)* |  | 12 | 179(22) | P<0.01 |
| *daf-2b(Δ)* control | 3* | 12 | 160(80) |  |
| *daf-2b(Δ)* |  | 12 | 177(63) | ns |
| *daf-2c(Δ)* control |  | 12 | 159(81) |  |
| *daf-2c(Δ)* |  | 12 | 182(58) | ns |

* Data shown in Figure
