# Supplementary material for: An alternatively spliced, non-signaling insulin receptor modulates insulin sensitivity via insulin peptide sequestration in C. elegans
Source: eLife. 2020 Feb 25;9:e49917. doi: 10.7554/eLife.49917 (PMC7041946; doi:10.7554/eLife.49917)
Supplement: Supplementary file 2. [file elife-49917-supp2.docx]

**List of primers used in this study.**

| Purpose | Primers |
| --- | --- |
| *daf-2* promoter | 1 CCCAAGCTTTATAACGTTCTTCCCCTCCCA  2 ATTACCGGTCGTTCTGTCTGCATTTACAAGTG |
| *daf-2* Exon 11-Exon 12 genomic - | 3 ATTACCGGTATGAGCACCCCCGCAAATCGACAAC  4 CGGGGTACCGGCGCATAAAGTGTGTAATGCTTCAAATTTCGT |
| TdTomato::*unc-54* 3’UTR | 5 CGGGGTACCGGTAATGGTGAGCAA  6 CGTACGGCCGACTAGTAGGAAACAGTTA |
| *daf-2* Exon 11-Exon 11.5 genomic - | 7 ATTACCGGTATGAGCACCCCCGCAAATCGACAAC  8 CGGGGTACCCCATCATGCATTACAGACATCATCAAATCATG |
| TdTomato | 9 CGGGGTACCGGTAATGGTGAGCAA  10 GTCGACTTACTTGTACAGCTCGTCCATGC |
| *daf-2* intron 11.5-exon 12::*unc-54* 3’ UTR | 11 ACGCGTCGACGCGCGAAATTTGAATTTTTCAAAAAAAATT  12 CGTACGGCCGACTAGTAGGAAACAGTTA |
| GFP + polylinker | 13 ATAGACCCTGCAGGACCGGTATAAGAATGCGGCCGCAATGAGTAAAGGAGAAGA ACTTTTCACTG  14 CTAGCTAGCCTATTTGTATAGTTCATCCATGCCATGT |
| *rab-3* promoter | 15 ACATGCATGCGAGCAGTGGACTGTCTATTTG  16 ATTACCGGTCTGAAAATAGGGCTACTGTAGAT |
| *myo-3* promoter | 17 CCCAAGCTTCCCGACAAAACATGAGTATTTC  18 ACCGGTTCTAGATGGATCTAGTGGTCGTG |
| *pges-1* promoter | 19 CAGTTTAAGCTTTTGGCATGAATACA  20 ATAGACCCTGCAGGCTGAATTCAAAGATAAGATATGTAATAG |
| *tag-335* promoter | 21 ATAGACCCTGCAGGGCGATCACCAGCAAGAAGAG  22 ATTACCGGTTTGATGGAAGTGGTGAAACAACC |
| *prgef-1* promoter | 23 ATAGACCCTGCAGGCGATAATCTCGTGACACTCGTTTC  24 ATTACCGGTCGTCGTCGTCGTCGATGC |
| *punc-122* promoter | 25 CCCAAGCTTTGTACGTTACATCTCATATACTCTGGGCAT  26 ATTACCGGTATTGTGAGCCCAATGAAGTAAAATTTCATGCT |
| *pdpy-7* promoter | 27 CCCAAGCTTAAAGTTTGGAGAAGTGATGATTG  28 ATAGACCCTGCAGGATCTGGAACAAAATGTAAGAATATT |
| *daf-2b cDNA* | 29 ATAGACCCTGCAGGACCGGTATAAGAATGCGGCCGCAATGACGAGAATGAATATT GTCAGATGT  30 CTAGCTAGCCTAATCATGCATTACAGACATCATCAA |
| C-terminal FLAG + *unc-54* 3’ UTR | 31 ATAAGAATGCGGCCGCGGATTACAAGGATGACGATGACAAGGACTATAAGGAC  32 CGTACGGCCGACTAGTAGGAAACAGTTA |
| Full length *daf-2b* cDNA without stop | 33 ATTACCGGTATGACGAGAATGAATATTGTCAGATGT  34 ATAAGAATGCGGCCGCATCATGCATTACAGACATCATCAA |
| *daf-2b* cDNA with C196Y mutation | 35 CGAAAAACGATACGGCCCAATCG  36 ACTATTGAGGCACACGGC |
| *daf-28* promoter | 37 ATAGACCCTGCAGGTAAATGAGCATAATTCATAGACTAACAAGTTTTG  38 ATTACCGGTGTTGAGATAGTTGTTGAGAGGAGACAGA |
| *daf-28* genomic DNA | 39 ATTACCGGTATGAACTGCAAGCTCATCGC  40 CGGGGTACCTTAAAGAAGCAAACGTGGGCAAC |
| *ins-6* genomic DNA | 41 ATTACCGGTATGAACTCTGTCTTTACTATCATCTTCGTTTTG  42 CGGGGTACCTCATGGACAACAAGCAGATCTTATG |
| *ins-18* promoter + genomic DNA | 43 ATAGACCCTGCAGGGATGCTTTTTAGAATGTCCGGAAAGTG  44 CGGGGTACCTTAAATTGGGGCACAGTAGGCAAG |
| Q5 SDM C-HA | 45 CCGGATTATGCGTAAGGGATCCAGACATGATAAGATACATTGATGAGTTTGGACA AAC  46 CACATCATACGGATACGCGGCCGCGGTACCTCG |
| Q5 SDM C-MYC | 47 CGAAGAAGACCTGTAAGGATCCAGACATGATAAGATACATTGATGAGTTTGGACA AACC  48 CTAATCAGTTTCTGTTCCGCGGCCGCGGTACCTCG |
| *daf-2b* cDNA | 49 GAAGATCTTATGACGAGAATGAATATTGTCAGAT  50 ATAAGAATGCGGCCGCATCATGCATTACAGACATCATCAA |
| *daf-2* cDNA Ex11-Ex11.5 | 51 AACACTTGTAAATGCAGACAGAACGACCGGTATGACGAGAATGAATATTGTC  52 ACTCGCATCGTGTCTAAAATCATATCAGCAAACTGTTC |
| *daf-2* cDNA Ex12-17 | 53 TGCTGATATGATTTTAGACACGATGCGAGTGAG  54 TGCGGAGCTCAGATATCAATACCATGGTACCTCAGACAAGTGGATGATGCTCATT ATC |
| *daf-2b* crRNA | 55 GATGTCTGTAATGCATGATT GUUUUAGAGCUAUGCUGUUUUG |
| *mScarlet* HDR template | 56 ATTTGCATGATTTGATGATGTCTGTAATGCATGATATGGTCTCCAAGGGAGAGGC  57 ATTCGAATTTTTTTTGAAAAATTCAAATTTCGCGCTTACTTGTAGAGCTCGTCCATT CCTC |
| *dpy-10* crRNA | 58 GCUACCAUAGGCACCACGAGGUUUUAGAGCUAUGCUGUUUUG |
| *dpy-10* repair | 59 CACTTGAACTTCAATACGGCAAGATGAGAATGACTGGAAACCGTACCGCATGCGG TGCCTATGGTAGCGGAGCTTCACATGGCTTCAGACCAACAGCCTAT |
| tracrRNA | 60 AACAGCAUAGCAAGUUAAAAUAAGGCUAGUCCGUUAUCAACUUGAAAAAGUGGC ACCGAGUCGGUGCUUUUUUU |
| *daf-2b::mScarlet* genotyping | 61 TGCATTTGAGAATAAGCTGTTGG  62 TTGATCGTCTCACTCGCATC |
| *daf-2b::mScarlet* sequencing | 63 GCGACATAAAAAATGATCCTG  64 CTTGTAGATGAGGGTTCCGTCCTCG  65 CGAATGTAATATGTACTGTAATTATTAAATATTAAATTCTCG |
| *daf-2* exon 11 crRNA | 66 TGGATGAGGTTTTAATGCCG GUUUUAGAGCUAUGCUGUUUUG |
| *daf-2* exon 12 crRNA | 67 GAATCGAGTCAGTGAAGAGT GUUUUAGAGCUAUGCUGUUUUG |
| *daf-2bc* HDR template | 68 ATTTGAGAATAAGCTGTTGGATGAGGTTTTAATGCCTCGAGACACGATGCGAGTG AGACGATCAATTGAAGACGCGAATCGAGTCAGTGAAGAGTTAGAAAAAGCTGAAAATTTGGGAAAAGCTC |
| *pha-1* crRNA | 69 ATGAATAACTTGATGAACAT GUUUUAGAGCUAUGCUGUUUUG |
| *pha-1* HDR template | 70 CAAAATACGAATCGAAGACTCAAAAAGAGTATGCTGTATGATTACAGATGTTCATC AAGTTATTCATAAATCATTGATAG |
| *daf-2bc(Δ)* genotyping | 71 TGCATTTGAGAATAAGCTGTTGG  72 TATGCCTGCTCCAAGCCTAT  73 CGCATAAAGTGTGTAATGCTTCAAAT |
| *daf-2bc(Δ)* sequencing | 74 GCCAGTCGATATTCCGTCAT  75 CGCATAAAGTGTGTAATGCTTCAAAT |
| MosSCI genotyping | 76 CGCTACTTACCGGAAACCAA  77 TTTCTCAGTTGTGATACGGTTTTT  78 CAATTCATCCCGGTTTCTGT  79 TCTGGCTCTGCTTCTTCGTT |
| Full length *daf-2b* cDNA | 80 ATGACGAGAATGAATATTGTCAGATGT  81 CAGACATCATCAAATCATGCAA |
| *daf-2b* cDNA exon 11 to 3’UTR | 82 GCCAGTCGATATTCCGTCAT  83 CACTTTTAATATGCTAAAATCTAATAAATAGAG |
