# Supplementary material for: An alternatively spliced, non-signaling insulin receptor modulates insulin sensitivity via insulin peptide sequestration in C. elegans
Source: eLife. 2020 Feb 25;9:e49917. doi: 10.7554/eLife.49917 (PMC7041946; doi:10.7554/eLife.49917)
Supplement: Supplementary file 3. [file elife-49917-supp3.docx]

**List of plasmids used in this study.**

| Name | Description | Comments |
| --- | --- | --- |
| pTG54 | *pdaf-2::daf-2a* cDNA | Gift from Rene Garcia at Texas A&M University |
| pKB1 | *prab-3::aak-2*(1-321 cDNA) tdTomato | Gift from Will Mair, Harvard School of Public Health |
| pPD95.75 | MCS::GFP::*unc54* UTR | Gift from Andrew Fire (Addgene kit # 1000000001). |
| pPD49.26 | MCS::MCS::*unc54* UTR | Gift from Andrew Fire (Addgene kit # 1000000001). |
| pHO4d-Cas9 | Cas9nlsHis6 Bacterial Expression construct | Gift from Michael Nonet (Addgene plasmid # 67881 ; http://n2t.net/addgene:67881 ; RRID:Addgene_67881) |
| pCFJ151 | *ttTi5605* targeting vector | Gift from Erik Jorgensen (Addgene plasmid # 19330 ; http://n2t.net/addgene:19330 ; RRID:Addgene_19330) |
| pCFJ601 | *Peft-3::*Mos1 transposase | Gift from Erik Jorgensen (Addgene plasmid # 34874 ; http://n2t.net/addgene:34874 ; RRID:Addgene_34874), |
| pMA122 | *peel-1* negative selection | Gift from Erik Jorgensen (Addgene plasmid # 34873 ; http://n2t.net/addgene:34873 ; RRID:Addgene_34873), |
| pGH8 | *pRAB-3::mCherry::unc-54utr* | Gift from Erik Jorgensen (Addgene plasmid # 19359 ; http://n2t.net/addgene:19359 ; RRID:Addgene_19359) |
| pCFJ90 | *Pmyo-2::mCherry::unc-54utr* | Gift from Erik Jorgensen (Addgene plasmid # 19327 ; http://n2t.net/addgene:19327 ; RRID:Addgene_19327) |
| pCFJ104 | *Pmyo-3::mCherry::unc-54* | Gift from Erik Jorgensen (Addgene plasmid # 19328 ; http://n2t.net/addgene:19328 ; RRID:Addgene_19328) |
| pBG-GY837 | *pCR8 mScarlet* | mScarlet in pCR8 |
| pMGL4 | pTG54 *pdaf-2::daf-2b* cDNA | *daf-2b* cDNA in pTG54 vector |
| pMGL83 | pBGY487 *pdaf-2::daf-2a/c::*tdTomato minigene | *daf-2a/c* splicing reporter |
| pMGL86 | pBGY487 *pdaf-2::daf-2b::*tdTomato minigene | *daf-2b* splicing reporter |
| pMGL119 | pPD49.26 *pdaf-2::daf-2b* cDNA | *daf-2b* expression from native promoter |
| pMGL120 | pPD49.26 *prab-3::daf-2b* cDNA | Pan-neuronal *daf-2b* expression |
| pMGL121 | pPD49.26 *pmyo-3::daf-2b* cDNA | Muscle *daf-2b* expression |
| pMGL123 | pPD49.26 *pges-1::daf-2b* cDNA | Intestinal *daf-2b* expression |
| pMGL150 | pPD49.26 *ptag-335::daf-2b* cDNA | Hypodermal *daf-2b* expression |
| pMGL212 | pPD49.26 *prgef-1::GFP* | Pan-neuronal GFP expression |
| pMGL153 | pPD49.26 *punc-122::GFP* | Coelomocyte GFP expression |
| pMGL116 | pPD49.26 *pdpy-7::GFP* | Hypodermal GFP expression |
| pMGL117 | pPD49.26 *pges-1::GFP* | Intestinal GFP expression |
| pMGL219 | pPD49.26 *pdaf-28::daf-28* | DAF-28 expression from native promoter |
| pMGL215 | pPD49.26 *prgef-1::ins-6* | INS-6 expression from the neuronal *rgef-1* promoter |
| pMGL221 | pPD49.26 *pins-18::ins-18* | INS-18 expression from native promoter |
| pMGL222 | pPD49.26 *prab-3::daf-2b::*FLAG | Neuronal *daf-2b* with C-terminal FLAG tag |
| pMGL223 | pPD49.26 *prab-3::daf-2b(*C196Y)::FLAG | Neuronal mutant *daf-2b* with C-terminal FLAG tag |
| pMGL166 | pPD49.26 *pdaf-2::daf-2c* | *daf-2c* expression from native promoter |
| pMGL170 | pCFJ151 *pdaf-2::daf-2b* | MosSCI targeting construct for *daf-2b* |
| pMGL171 | pCFJ151 *pdaf-2::daf-2c* | MosSCI targeting construct for *daf-2c* |
| pMGL133 | pCMV *daf-2b::*HA-C | *daf-2b* with C-terminal HA tag for cell expression |
| pMGL134 | pCMV *daf-2b::*Myc-C | *daf-2b* with C-terminal myc tag for cell expression |
| pMGL135 | pCMV *daf-2a::*HA-C | *daf-2a* with C-terminal HA tag for cell expression |
| pMGL136 | pCMV *daf-2a::*Myc-C | *daf-2a* with C-terminal myc tag for cell expression |
