# Supplementary material for: An alternatively spliced, non-signaling insulin receptor modulates insulin sensitivity via insulin peptide sequestration in C. elegans
Source: eLife. 2020 Feb 25;9:e49917. doi: 10.7554/eLife.49917 (PMC7041946; doi:10.7554/eLife.49917)
Supplement: Supplementary file 4. [file elife-49917-supp4.docx]

**List of *C. elegans* strains used in this study.**

| **Strain** | **Genotype** | **Source** | **Description** |
| --- | --- | --- | --- |
| N2 | N2 Bristol | CGC |  |
| DR1572 | *daf-2(e1368)* | CGC |  |
| JT709 | *pdk-1(sa709)* | CGC |  |
| EG6699 | *ttTi5605; unc-119(ed3); oxEx1578* | CGC |  |
| GE24 | *pha-1(e2123)* | CGC |  |
| MGL262 | *jluEx130[daf-2p::DAF-2a/cexon-12::tdTomato + rol-6(+)].* | This study | *daf-2a/c* splicing reporter |
| MGL263 | *jluEx131 [daf-2p::DAF-2bexon-11.5::tdTomato + rol-6(+)]* | This study | *daf-2b* splicing reporter |
| MGL264 | *jluIs15[daf-2p::DAF-2bexon-11.5::tdTomato + rol-6(+)]* | This study | Integrated *daf-2b* splicing reporter |
| MGL265 | *jluIs16[daf-2p::DAF-2bexon-11.5::tdTomato + rol-6(+)]* |  |  |
| MGL360 | *jluEx176[daf-2p::DAF-2a/cexon-12::tdTomato + rgef-1p::GFP + rol-6(+)].* | This study | *daf-2a/c* splicing reporter + neuronal GFP |
| MGL361 | *jluEx177[daf-2p::DAF-2a/cexon-12::tdTomato + dpy-7p::GFP + rol-6(+)].* | This study | *daf-2a/c* splicing reporter + hypodermal GFP |
| MGL362 | *jluEx178[daf-2p::DAF-2a/cexon-12::tdTomato + ges-1p::GFP + rol-6(+)].* | This study | *daf-2a/c* splicing reporter + intestinal GFP |
| MGL363 | *jluIs15; jluEx179[rgef-1p::GFP]* | This study | *daf-2b* splicing reporter + neuronal GFP |
| MGL364 | *jluIs15; jluEx180[dpy-7p::GFP]* | This study | *daf-2b* splicing reporter + hypodermal GFP |
| MGL365 | *jluIs15; jluEx181[ges-1p::GFP]* | This study | *daf-2b* splicing reporter + intestinal GFP |
| MGL366 | *jluIs15; jluEx182[unc-122p::GFP]* | This study | *daf-2b* splicing reporter + coelomocyte GFP |
| MGL367 | *daf-2(jlu2[daf-2b::mScarlet])* | This study | *daf-2b::mScarlet* CRISPR Knock-in |
| MGL368 | *daf-2(jlu3[daf-2b::mScarlet])* |  |  |
| MGL369 | *daf-2(jlu2[daf-2b::mScarlet]); jluEx183[rgef-1p::GFP]* | This study | *daf-2b::mScarlet* CRISPR Knock-in + neuronal GFP expression |
| MGL370 | *daf-2(jlu2[daf-2b::mScarlet]); jluEx184[unc-122p::GFP]* | This study | *daf-2b::mScarlet* CRISPR Knock-in + coelomocyte GFP expression |
| MGL266 | *jluEx132[daf-2p::DAF-2B + myo-2p::tdTomato]* | This study | Native DAF-2B overexpresser |
| MGL267 | *jluEx133[daf-2p::DAF-2B + myo-2p::tdTomato]* |  |  |
| MGL268 | *jluEx134[daf-2p::DAF-2B + myo-2p::tdTomato]* |  |  |
| MGL269 | *jluEx135[ges-1p::DAF-2B + myo-2p::tdTomato]* | This study | Intestinal DAF-2B overexpresser |
| MGL270 | *jluEx136[ges-1p::DAF-2B + myo-2p::tdTomato]* |  |  |
| MGL271 | *jluEx137[ges-1p::DAF-2B + myo-2p::tdTomato]* |  |  |
| MGL272 | *jluEx138[myo-3p::DAF-2B + myo-2p::tdTomato]* | This study | Muscle DAF-2B overexpresser |
| MGL273 | *jluEx139[myo-3p::DAF-2B + myo-2p::tdTomato]* |  |  |
| MGL274 | *jluEx140[myo-3p::DAF-2B + myo-2p::tdTomato]* |  |  |
| MGL275 | *jluEx141[rab-3p::DAF-2B + myo-2p::tdTomato]* | This study | Neuronal DAF-2B overexpresser |
| MGL276 | *jluEx142[rab-3p::DAF-2B + myo-2p::tdTomato]* |  |  |
| MGL277 | *jluEx143[rab-3p::DAF-2B + myo-2p::tdTomato]* |  |  |
| MGL278 | *jluEx144[tag-335p::DAF-2B + myo-2p::tdTomato]* | This study | Hypodermal DAF-2B overexpresser |
| MGL279 | *jluEx145[tag-335p::DAF-2B + myo-2p::tdTomato]* |  |  |
| MGL281 | *daf-2(e1368); jluEx132[daf-2p::DAF-2B + myo-2p::tdTomato]* | This study | *daf-2(e1368)* + native DAF-2B overexpresser |
| MGL282 | *daf-2(e1368); jluEx133[daf-2p::DAF-2B + myo-2p::tdTomato]* |  |  |
| MGL283 | *daf-2(e1368); jluEx134[daf-2p::DAF-2B + myo-2p::tdTomato]* |  |  |
| MGL284 | *daf-2(e1368); jluEx135[ges-1p::DAF-2B + myo-2p::tdTomato]* | This study | *daf-2(e1368)* + intestinal DAF-2B overexpresser |
| MGL285 | *daf-2(e1368); jluEx136[ges-1p::DAF-2B + myo-2p::tdTomato]* |  |  |
| MGL286 | *daf-2(e1368); jluEx137[ges-1p::DAF-2B + myo-2p::tdTomato]* |  |  |
| MGL287 | *daf-2(e1368); jluEx138[myo-3p::DAF-2B + myo-2p::tdTomato]* | This study | *daf-2(e1368)* + muscle DAF-2B overexpresser |
| MGL288 | *daf-2(e1368); jluEx139[myo-3p::DAF-2B + myo-2p::tdTomato]* |  |  |
| MGL289 | *daf-2(e1368); jluEx140[myo-3p::DAF-2B + myo-2p::tdTomato]* |  |  |
| MGL290 | *daf-2(e1368); jluEx141[rab-3p::DAF-2B + myo-2p::tdTomato]* | This study | *daf-2(e1368)* + neuronal DAF-2B overexpresser |
| MGL291 | *daf-2(e1368); jluEx142[rab-3p::DAF-2B + myo-2p::tdTomato* |  |  |
| MGL292 | *daf-2(e1368); jluEx143[rab-3p::DAF-2B + myo-2p::tdTomato]* |  |  |
| MGL293 | *daf-2(e1368); jluEx144[tag-335p::DAF-2B + myo-2p::tdTomato]* | This study | *daf-2(e1368)* + hypodermal DAF-2B overexpresser |
| MGL294 | *daf-2(e1368); jluEx145[tag-335p::DAF-2B + myo-2p::tdTomato]* |  |  |
| MGL295 | *jluSi1[daf-2p::DAF-2B + unc-119(+)]* | This study | *daf-2b(+) -* Mos SCI *daf-2b* cDNA at |
| MGL296 | *jluSi2[daf-2p::DAF-2B + unc-119(+)]* |  | [*ttTi5605*](https://cgc.umn.edu/variation/1888667) *II* |
| MGL297 | *jluSi3[daf-2p::DAF-2C + unc-119(+)]* | This study | *daf-2c(+) -* Mos SCI *daf-2c* cDNA at |
| MGL298 | *jluSi4[daf-2p::DAF-2C + unc-119(+)]* |  | [*ttTi5605*](https://cgc.umn.edu/variation/1888667) *II* |
| MGL299 | *daf-2(jlu1)* | This study | *daf-2bc(Δ)* |
| MGL300 | *jluSi1[daf-2p::DAF-2B + unc-119(+)]; daf-2(jlu1)* | This study | *daf-2c(Δ) - daf-2bc(Δ) + daf-2b(+)* |
| MGL301 | *jluSi2[daf-2p::DAF-2B + unc-119(+)]; daf-2(jlu1)* |  |  |
| MGL302 | *jluSi3[daf-2p::DAF-2C + unc-119(+)]; daf-2(jlu1)* | This study | *daf-2b(Δ) - daf-2bc(Δ) + daf-2c(+)* |
| MGL303 | *jluSi4[daf-2p::DAF-2C + unc-119(+)]; daf-2(jlu1)* |  |  |
| MGL304 | *jluSi1[daf-2p::DAF-2B + unc-119(+)]; pdk-1(sa709)* | This study | *pdk-1(sa709) + daf-2b(+)* |
| MGL305 | *jluSi2[daf-2p::DAF-2B + unc-119(+)];pdk-1(sa709)* |  |  |
| MGL306 | *jluSi3[daf-2p::DAF-2C + unc-119(+)]; pdk-1(sa709)* | This study | *pdk-1(sa709) + daf-2c(+)* |
| MGL307 | *jluSi4[daf-2p::DAF-2C + unc-119(+)];pdk-1(sa709)* |  |  |
| MGL308 | *daf-2(jlu1); pdk-1(sa709)* | This study | *pdk-1(sa709) + daf-2bc(Δ)* |
| MGL309 | *jluSi1[daf-2p::DAF-2B + unc-119(+)];daf-2(jlu1); pdk-1(sa709)* | This study | *pdk-1(sa709) + daf-2c(Δ)* |
| MGL310 | *jluSi2[daf-2p::DAF-2B + unc-119(+)]; daf-2(jlu1); pdk-1(sa709)* |  |  |
| MGL311 | *jluSi3[daf-2p::DAF-2C + unc-119(+)]; daf-2(jlu1); pdk-1(sa709)* | This study | *pdk-1(sa709) + daf-2b(Δ)* |
| MGL312 | *jluSi4[daf-2p::DAF-2C + unc-119(+)]; daf-2(jlu1); pdk-1(sa709)* |  |  |
| MGL347 | *jluIs17[rab-3p::DAF-2B + myo-2p::tdTomato];* | This study | Integrated neuronal DAF-2B overexpresser |
| MGL313 | *jluIs17[rab-3p::DAF-2B + myo-2p::tdTomato]; daf-2(e1368)* | This study | *daf-2(e1368)* + integrated neuronal DAF-2B overexpresser |
| MGL314 | *daf-2(e1368); jluEx146[daf-28p::DAF-28 + myo-3p::GFP]* | This study | *daf-2(e1368) +* native DAF-28 overexpresser |
| MGL315 | *daf-2(e1368); jluEx147[daf-28p::DAF-28 + myo-3p::GFP]* |  |  |
| MGL316 | *daf-2(e1368); jluEx148[daf-28p::DAF-28 + myo-3p::GFP]* |  |  |
| MGL317 | *daf-2(e1368); jluEx149[daf-28p::DAF-28 + myo-3p::GFP]* |  |  |
| MGL318 | *daf-2(e1368); jluEx150[daf-28p::DAF-28 + myo-3p::GFP]* |  |  |
| MGL319 | *daf-2(e1368); jluEx151[daf-28p::DAF-28 + myo-3p::GFP]* |  |  |
| MGL320 | *daf-2(e1368); jluEx146[daf-28p::DAF-28 + myo-3p::GFP]; jluIs17* | This study | *daf-2(e1368)* + native DAF-28 overexpresser + integrated neuronal DAF-2B overexpresser |
| MGL321 | *daf-2(e1368); jluEx147[daf-28p::DAF-28 + myo-3p::GFP]; jluIs17* |  |  |
| MGL322 | *daf-2(e1368); jluEx148[daf-28p::DAF-28 + myo-3p::GFP]; jluIs17* |  |  |
| MGL323 | *daf-2(e1368); jluEx149[daf-28p::DAF-28 + myo-3p::GFP]; jluIs17* |  |  |
| MGL324 | *daf-2(e1368); jluEx150[daf-28p::DAF-28 + myo-3p::GFP]; jluIs17* |  |  |
| MGL325 | *daf-2(e1368); jluEx151[daf-28p::DAF-28 + myo-3p::GFP]; jluIs17* |  |  |
| MGL326 | *daf-2(e1368); jluEx152[rgef-1p::INS-6 + myo-3p::GFP]* | This study | *daf-2(e1368)* + neuronal INS-6 overexpresser |
| MGL327 | *daf-2(e1368); jluEx153[rgef-1p::INS-6 + myo-3p::GFP]* |  |  |
| MGL328 | *daf-2(e1368); jluEx154[rgef-1p::INS-6 + myo-3p::GFP]* |  |  |
| MGL329 | *daf-2(e1368); jluEx155[rgef-1p::INS-6 + myo-3p::GFP]* |  |  |
| MGL330 | *daf-2(e1368); jluEx156[rgef-1p::INS-6 + myo-3p::GFP]* |  |  |
| MGL331 | *daf-2(e1368); jluEx157[rgef-1p::INS-6 + myo-3p::GFP]* |  |  |
| MGL332 | *daf-2(e1368); jluEx155[rgef-1p::INS-6 + myo-3p::GFP]; jluIs17* | This study | *daf-2(e1368) +* neuronal INS-6 overexpresser + integrated neuronal DAF-2B overexpresser |
| MGL333 | *daf-2(e1368); jluEx156[rgef-1p::INS-6 + myo-3p::GFP]; jluIs17* |  |  |
| MGL334 | *daf-2(e1368); jluEx157[rgef-1p::INS-6 + myo-3p::GFP]; jluIs17* |  |  |
| MGL335 | *daf-2(e1368); jluEx158[rab-3p::DAF-2B::FLAG]* | This study | *daf-2(e1368) +* DAF-2B::FLAG overexpresser |
| MGL336 | *daf-2(e1368); jluEx159[rab-3p::DAF-2B::FLAG]* |  |  |
| MGL337 | *daf-2(e1368); jluEx160[rab-3p::DAF-2B::FLAG]* |  |  |
| MGL338 | *daf-2(e1368); jluEx161[rab-3p::DAF-2B::FLAG]* |  |  |
| MGL339 | *daf-2(e1368); jluEx162[rab-3p::DAF-2B::FLAG]* |  |  |
| MGL340 | *daf-2(e1368); jluEx163[rab-3p::DAF-2B::FLAG]* |  |  |
| MGL341 | *daf-2(e1368); jluEx164[rab-3p::DAF-2B(C196Y)::FLAG]* | This study | *daf-2(e1368) +* mutant DAF-2B::FLAG overexpresser |
| MGL342 | *daf-2(e1368); jluEx165[rab-3p::DAF-2B(C196Y)::FLAG]* |  |  |
| MGL343 | *daf-2(e1368); jluEx166[rab-3p::DAF-2B(C196Y)::FLAG]* |  |  |
| MGL344 | *daf-2(e1368); jluEx167[rab-3p::DAF-2B(C196Y)::FLAG]* |  |  |
| MGL345 | *daf-2(e1368); jluEx168[rab-3p::DAF-2B(C196Y)::FLAG]* |  |  |
| MGL346 | *daf-2(e1368); jluEx169[rab-3p::DAF-2B(C196Y)::FLAG]* |  |  |
| MGL348 | *jluEx170[ins-18p::INS-18 + myo-3p::GFP]* | This study | Native INS-18 overexpresser |
| MGL349 | *jluEx171[ins-18p::INS-18 + myo-3p::GFP]* |  |  |
| MGL350 | *jluEx172[ins-18p::INS-18 + myo-3p::GFP]* |  |  |
| MGL351 | *jluEx173[ins-18p::INS-18 + myo-3p::GFP]* |  |  |
| MGL352 | *jluEx174[ins-18p::INS-18 + myo-3p::GFP]* |  |  |
| MGL353 | *jluEx175[ins-18p::INS-18 + myo-3p::GFP]* |  |  |
| MGL354 | *jluEx170[ins-18p::INS-18 + myo-3p::GFP]; jluIs17* | This study | Native INS-18 overexpresser + integrated neuronal DAF-2B overexpresser |
| MGL355 | *jluEx171[ins-18p::INS-18 + myo-3p::GFP]; jluIs17* |  |  |
| MGL356 | *jluEx172[ins-18p::INS-18 + myo-3p::GFP]; jluIs17* |  |  |
| MGL357 | *jluEx173[ins-18p::INS-18 + myo-3p::GFP]; jluIs17* |  |  |
| MGL358 | *jluEx174[ins-18p::INS-18 + myo-3p::GFP]; jluIs17* |  |  |
| MGL359 | *jluEx175[ins-18p::INS-18 + myo-3p::GFP]; jluIs17* |  |  |
